# Beyond the Norm: Navigating a Unique Papillary Carcinoma Journey in Breast Cancer

**DOI:** 10.7759/cureus.59795

**Published:** 2024-05-07

**Authors:** Shakti Sagar, K.M. Hiwale, Pravin Gadkari, Anil K Agrawal, Suhit Naseri, Simran Khan

**Affiliations:** 1 Pathology, Jawaharlal Nehru Medical College, Datta Meghe Institute of Higher Education and Research, Wardha, IND

**Keywords:** immunohistochemistry, lymph node, breast tumor, simple mastectomy, invasive papillary carcinoma

## Abstract

The presence of papillary structures inside the tumor is a unique and uncommon characteristic of breast cancer, and it is known as papillary carcinoma. In contrast to other forms of breast cancer, this variant usually manifests as a well-defined mass in imaging investigations and is frequently linked to a good prognosis. We present a case of a 72-year-old female with papillary carcinoma of the breast identified after presenting with a palpable breast lump. Following a left simple mastectomy and adjuvant treatment, the presence of papillary structures inside the tumor was verified by a histopathological study. Understanding the clinical and pathological characteristics of breast papillary carcinoma is crucial for precise diagnosis and suitable therapy strategizing. More research is required to further understand the molecular traits and best practices for treating this uncommon subtype of breast cancer.

## Introduction

Compared to other types of breast cancer, invasive papillary carcinoma is an uncommon malignant tumor with a significantly higher chance of survival. Papillary carcinoma of the breast is a rare entity, accounting for less than 1-2% of all invasive carcinoma cases [1]. It is predominantly seen in postmenopausal women and has a good prognosis [2]. Even though axillary node metastases are uncommon, mastectomy and axillary node dissection are common forms of therapy. Between 22 and 34% of instances demonstrate bloody nipple discharge as a presenting symptom; it can also manifest as a palpable lump. Grossly, it appears to be a grayish-white, ill-defined, ulceroproliferative mass. From a histopathological perspective, it consists of papillae generated by malignant epithelial cells closely associated with fibrovascular centers within a papillary architecture. It is often seen as a circumscribed mass, with cystic areas [3]. We describe an uncommon kind of breast tumor that presented preoperative diagnosis challenges. Despite its infrequency, its distinct clinical and pathological characteristics warrant attention for an accurate diagnosis and appropriate management. We discuss a case of breast papillary cancer in this report, emphasizing the histological characteristics, clinical symptoms, diagnostic workup, and treatment options. Our primary objective is to add to the existing body of knowledge on this uncommon entity by illuminating its clinical presentations and recommended courses of action.

## Case presentation

**Figure 1:**
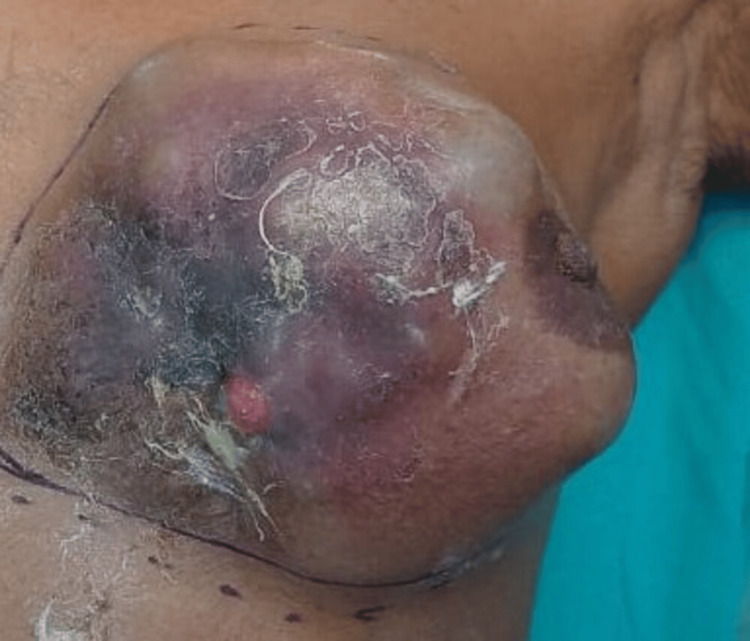
Clinical photomicrograph of the patient

A 72-year-old woman presented to the outpatient department for surgery with a five-year-old breast mass. The physical examination revealed a palpable lump of size 10 x 10 cm, which was large, nodular, hard in consistency, non-mobile, tender, fixed to the skin, and associated with areas of inflammation, congestion, and scaling of the skin. It was also associated with pain and pus discharge, which was insidious in onset and progressive in nature (Figure 1).

**Figure 2:**
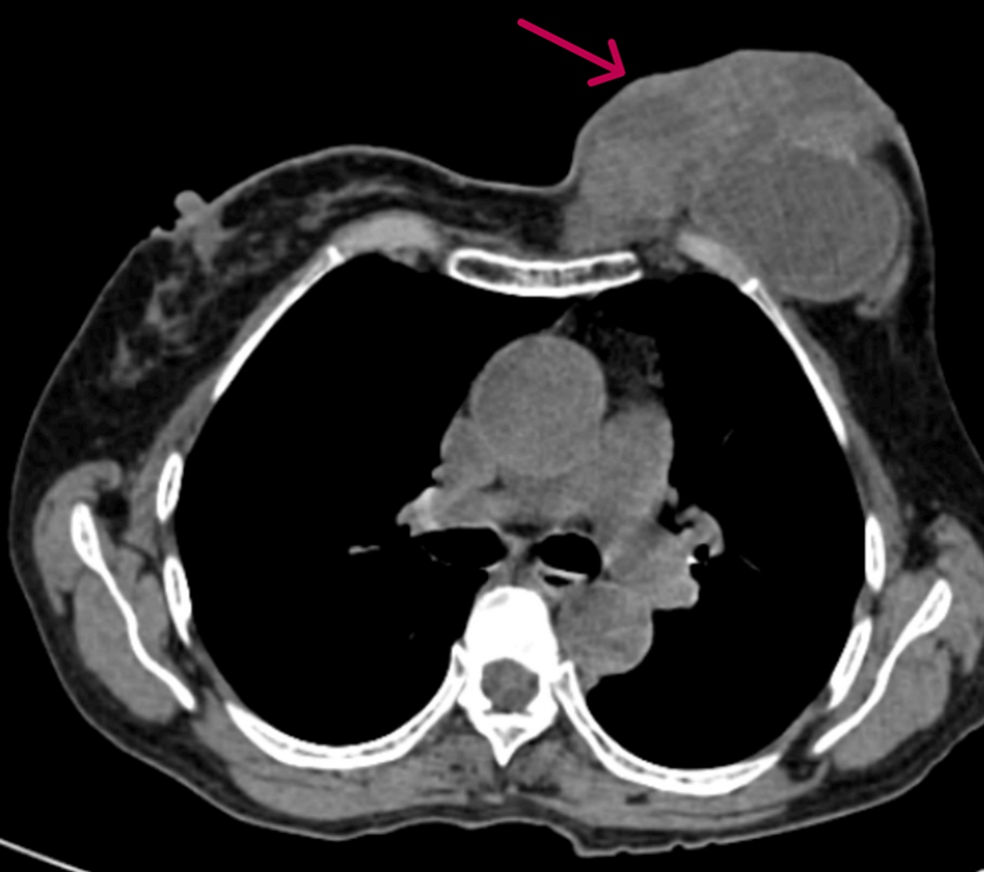
Contrast-enhanced CT of the thorax of the patient The image shows a heterogeneously enhancing, multilobulated, ulceroproliferative lesion in the left breast parenchyma with adjacent fat stranding and multiple non-enhancing areas of central necrosis CT: computed tomography

**Figure 3:**
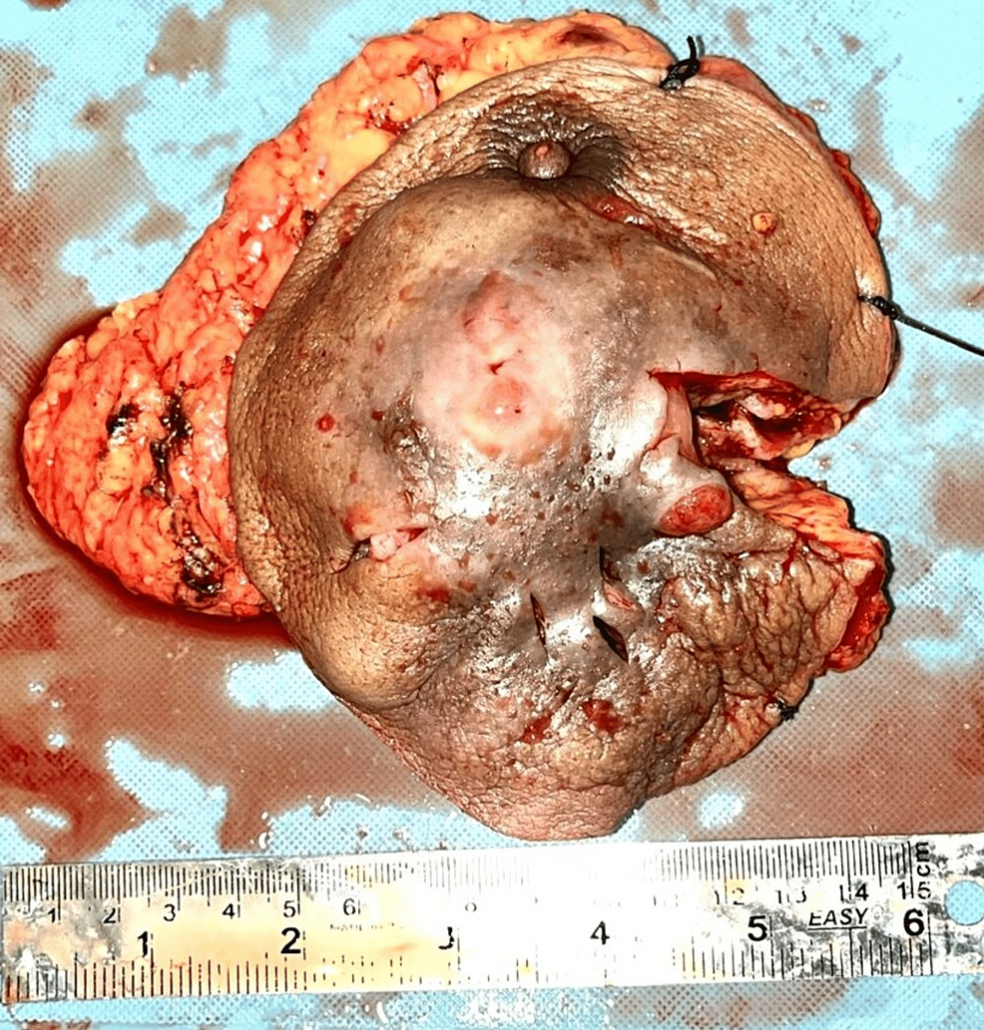
Postoperative specimen of the simple mastectomy of the left breast

The patient underwent an ultrasound examination. Ultrasonography (USG) breast imaging suggested phyllodes or neoplastic lesions in the left breast. Contrast-enhanced CT (CECT) thorax showed a heterogeneously enhancing, multilobulated, ulceroproliferative lesion in the left breast parenchyma with adjacent fat stranding and multiple non-enhancing areas of central necrosis measuring approximately 11 x 8.8 x 7 cm, with loss of fat planes with underlying pectoralis major and minor muscles (Figure 2), and bilateral metastatic axillary lymphadenopathy. There was no evidence of bony erosions of underlying ribs. A core-cut biopsy from the left breast was performed, which was inadequate to suggest a malignancy. Since clinical, sonographical, and CECT thorax findings were suggestive of breast cancer with axillary metastasis, a simple mastectomy was performed, followed by axillary lymph node dissection, and the specimen was sent for histopathology analysis (Figure 3).

**Figure 4:**
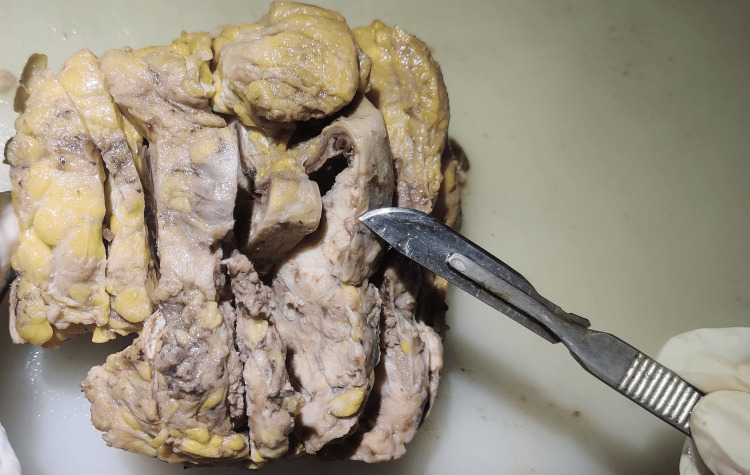
Gross photograph of simple mastectomy specimen of the left breast

We received a mastectomy specimen measuring 13 x 11 x 4 cm. The overlying skin showed discoloration. Satellite nodules measuring 1 x 0.5 cm were present. Grossly, the skin was affected by the tumor. A single, solid, grayish-white, ill-defined ulceroproliferative growth measuring 6 x 5.5 x 4.5 cm was identified in the lower quadrant majorly (Figure 4).

**Figure 5:**
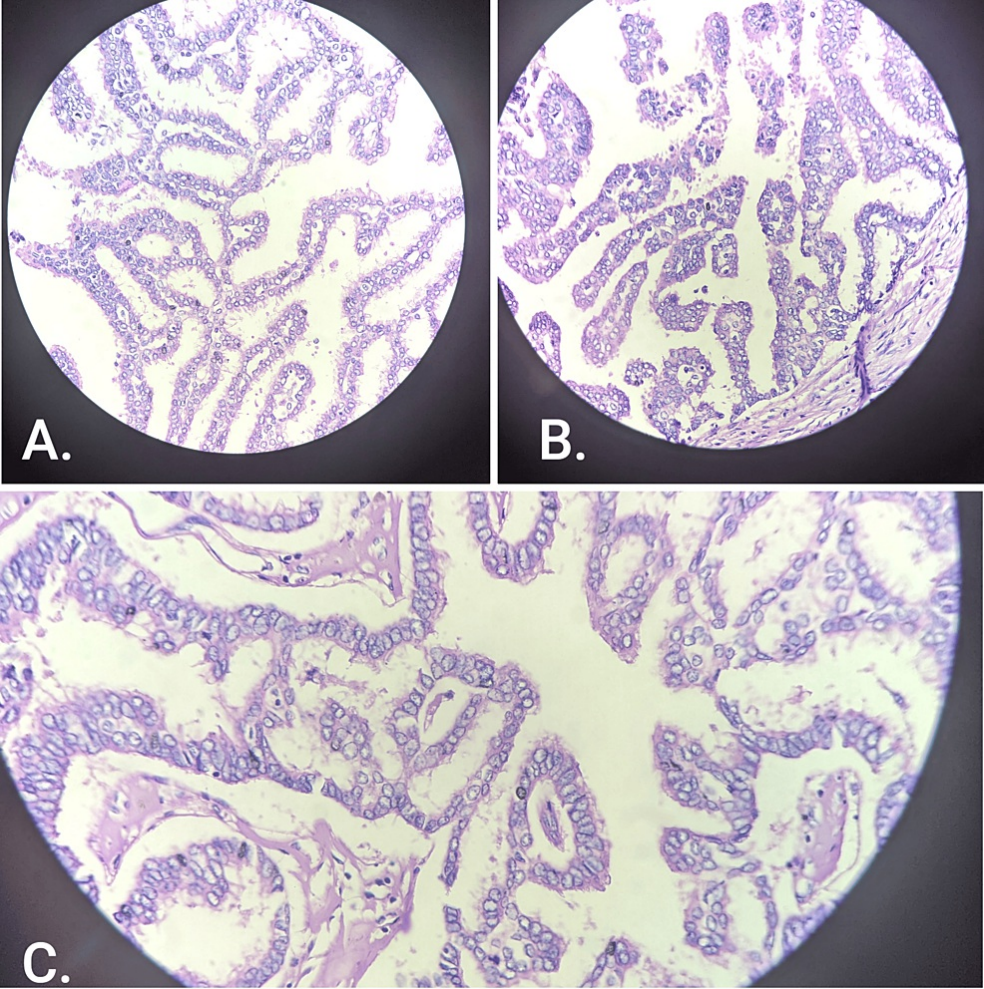
Section showing true papillae lined by neoplastic cells, columnar in shape, prominent nucleoli, loss of polarity, and abnormal mitosis invading the underlying stroma Haematoxylin and eosin (H&E) stain, 40x magnification

A cystically dilated space measuring 3.5 x 3 cm was identified within the tumor parenchyma. On microscopy, the section showed true papillae lined by neoplastic cells, columnar in shape, prominent nucleoli, irregular clump chromatin, irregular nuclear membrane, loss of polarity, and abnormal mitosis invading the underlying stroma and peritumoral fat. Papillae lacked myoepithelial cells (Figure 5).

**Figure 6:**
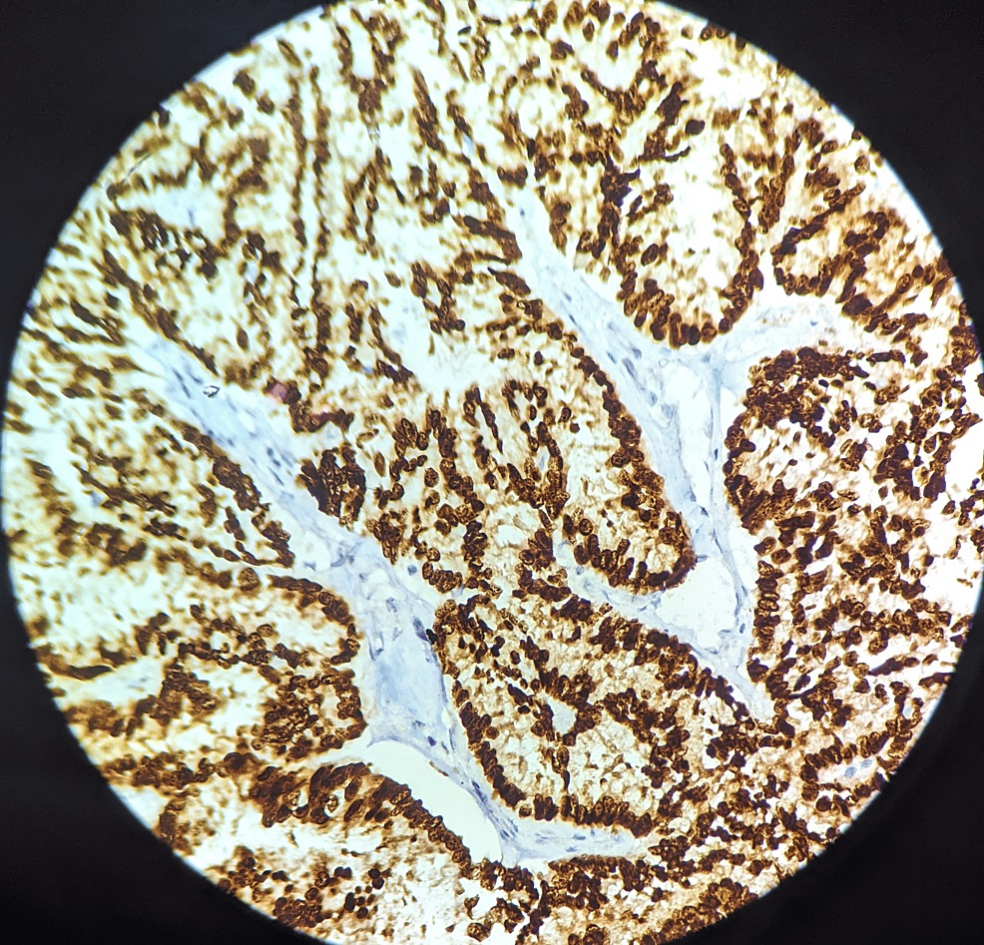
Estrogen receptor immunohistochemistry revealed a strong positive immunological staining DAB, 40x magnification

**Figure 7:**
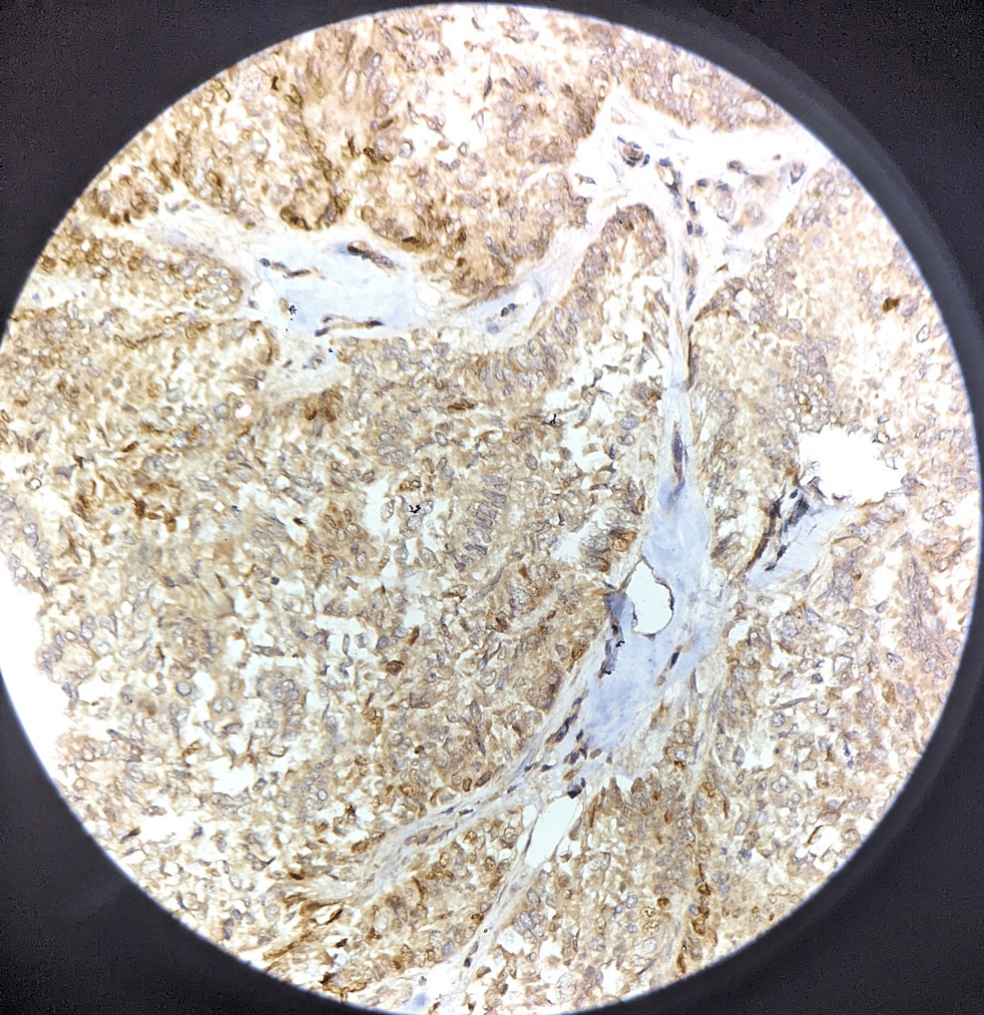
Progesterone receptor immunohistochemistry revealed a generalized positive immunological staining DAB, 40x magnification

**Figure 8:**
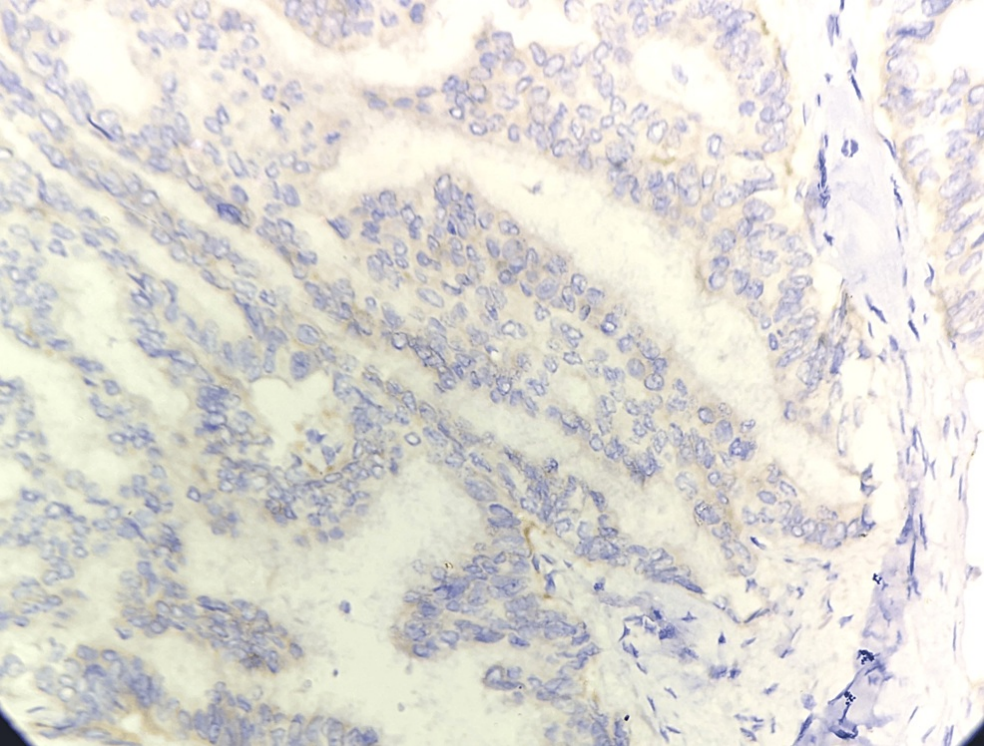
Human epidermal receptor-2 protein immunohistochemistry revealed a negative immunological staining DAB, 40x magnification

A section of the skin was positive for infiltration by malignant cells, possibly metastasis from the thyroid, ovary, and lung. However, the thyroid, ovary, and lung scans were normal. We also received axillary lymph nodes in a separate container. A total of nine lymph nodes were identified. Of them, five lymph nodes were positive for infiltration by malignant cells on histopathology. The remaining four lymph nodes showed histological features suggestive of reactive lymphadenitis. Progesterone receptor (PR), estrogen receptor (ER), human epidermal growth factor receptor 2 (HER2) Neu, and ki-67 were all immunohistochemically stained. The findings showed that ER was positive (score of 8), PR was positive (score of 7), HER2 Neu was negative, and ki-67 was less than 14% (Figures 6-8). Immunohistochemistry for TTF1, Napsin A, and PAX8 was negative.

Invasive papillary cancer of the left breast, luminal A type, was the final diagnosis. According to the College of American Pathologists (CAP) guidelines, the stage was pT4b N2b Mx. Letrozole hormone treatment following surgery, cyclophosphamide chemotherapy, and radiation to the left upper and lower clavicles as well as the left chest wall were started. There was no evidence of distant metastasis or recurrence during a 12-month follow-up period.

## Discussion

Papillary carcinoma of the breast is a rare type of invasive tumor, predominantly seen in the postmenopause age group of women [4]. Compared to other breast carcinomas, it has a better prognosis. Papillary lesions of the breast comprise a morphologically heterogeneous group of lesions and present challenges in differentiating benign from malignant lesions [5]. Papillary lesions comprise <10% of all benign lesions of the breast, while the papillary lesions account for <0.5-2% of all malignant lesions of the breast [6]. Clinically, it often manifests as a discharge from the nipples or a breast lump. Unlike other ductal carcinomas, which are hard and fixed, the lump in question is typically firm and movable. The incidence of malignancy at surgical excision for papillary lesions found on percutaneous core biopsy ranges from 17 to 34% in some studies [7, ]. Because the risk of malignancy upgrades is high, all papillary lesions should undergo excisional biopsies.

Pathologically, papillary cancer develops in the form of fronds with no myoepithelial layer and a fibrovascular core. Malignant papillary neoplasm of the breast includes many differential diagnoses, such as ductal carcinoma in situ (DCIS) arising in intraductal papilloma, papillary DCIS, encapsulated papillary carcinoma, and solid papillary carcinoma [6]. Other differential diagnoses like metastasis from thyroid, ovary, lung, and carcinoma of unknown primary should be ruled out. All malignant papillary lesions of the breast lack an intact myoepithelial cell layer within the papillae or at the periphery of the tumor, which is an important feature allowing distinction from benign intraductal papillomas [9].

When diagnosing invasive tumors, immunohistochemistry is a highly helpful tool for evaluating myoepithelial cells and basement membranes. Invasive tumors lack myoepithelial cells. Numerous well-known myoepithelial markers, with varying sensitivities and specificities, include S-100, calponin, CD 1O, alpha-smooth muscle actin, smooth muscle myosin heavy chain, P63, and high molecular weight cytokeratin. Myosin is the smooth muscle, and P63 is more particular. A special myoepithelial marker, P63, stains the cell muscles only [10]. Furthermore, the neoplastic cell population has substantial positivity for the estrogen and progesterone receptors but lacks the production of high molecular weight keratin.

A combination of surgery, radiation, chemotherapy, hormone treatment, and/or HER2 protein-targeting therapy is frequently used to treat papillary carcinoma. El Sheikh et al. state that if an intraductal mass fills the duct and expands beyond it at a distance greater than 1.5 cm from the nipple, cancer is more likely to occur. In our case, the mass was filling the duct as well as extending outside the duct and was 2 cm away from the nipple, thereby indicating the malignant nature of the mass [11]. The prognosis is usually excellent, with one study reporting disease-free 10-year survival rates of 91% and overall survival at 10 years of 100% [12].

## Conclusions

Invasive papillary carcinoma of the breast is a distinct and rather uncommon subtype of breast cancer that poses difficulties in both diagnosis and treatment. In this case report, we have highlighted the patient's clinical presentation, imaging results, histological characteristics, and course of treatment for invasive breast papillary cancer. The patient received effective treatment with surgery and adjuvant therapy using a multidisciplinary strategy involving medical oncologists, pathologists, radiologists, and surgical oncologists, with a positive outcome. It is crucial to raise awareness and understanding of this subtype of the disease to keep improving patient treatment, outcomes, and quality of life for those with invasive papillary carcinoma of the breast.
